# Cu(I) Coordination Complex Precursor for Randomized CuO*_x_* Microarray Loaded on Carbon Nanofiber with Excellent Electrocatalytic Performance for Electrochemical Glucose Detection

**DOI:** 10.3390/s19245353

**Published:** 2019-12-04

**Authors:** Sorina Motoc, Carmen Cretu, Otilia Costisor, Anamaria Baciu, Florica Manea, Elisabeta I. Szerb

**Affiliations:** 1 “Coriolan Dragulescu” Institute of Chemistry, Romanian Academy, 24 Mihai Viteazu Bvd., 300223 Timisoara, Romania; sorinamotoc@yahoo.com (S.M.); cretucami@yahoo.com (C.C.); ocostisor@acad-icht.tm.edu.ro (O.C.); 2 Department of Applied Chemsitry and Engineering of Inorganic Compounds and Environment, Politehnica University of Timisoara, 2 Victoriei Square, 300006 Timisoara, Romania; anamaria.baciu@upt.ro

**Keywords:** Cu(I) coordination complex, non-enzymatic glucose sensor, hybrid electrode, randomized copper oxide microarray, voltammetric and amperometric detection

## Abstract

A homoleptic ionic Cu(I) coordination complex that was based on 2,2′-biquinoline ligand functionalized with long alkyl chains (Cu(I)–C18) was used as a precursor to modify a carbon nanofiber paste electrode (Cu–C18/CNF). Randomized copper oxide microelectrode arrays dispersed within carbon nanofiber paste (CuO*_x_*/CNF) were obtained by electrochemical treatment of Cu–C18/CNF while using cyclic voltammetry (CV). The CuO*_x_*/CNF exhibited high electrocatalytic activity towards glucose oxidation at +0.6 V and +1.2 V vs. Ag/AgCl. Infrared Spectroscopy (FTIR) and scanning electron microscopy (SEM) characterized the electrodes composition. Cyclic voltammetry (CV), square wave-voltammetry (SWV), and multiple-pulsed amperometry (MPA) techniques provided optimized conditions for glucose oxidation and detection. A preconcentration step that involved 10 minutes accumulation at open circuit potential before SWV running led to the lowest limit of detection and the highest sensitivity for glucose detection (5419.77 µA·mM^−1^·cm^−2^ at + 1.1 V vs. Ag/AgCl) vs. Cu-based electrodes reported to date in literature.

## 1. Introduction

The rapid determination of glucose concentration has attracted increasing interest in various fields, e.g., clinical diagnosis, food industry, wastewater treatment, and sustainable fuel cells [1,2]. Electrochemical techniques exhibit great potential for the development of next-generation glucose detection sensors due to their advantages, e.g., high sensitivity and selectivity, accuracy, simple instrumentation, low cost, and excellent compatibility with miniaturization. Thus far, commercial glucose sensors are mainly built on enzyme-based electrochemical sensors. Despite good sensitivity and selectivity, their drawbacks related to low reproducibility, complexity of enzyme immobilization process, activity vulnerability, and high cost have been limited their large-scale practical applications [3,4]. Much effort has been devoted to the design and exploitation of non-enzymatic sensors based on direct electrocatalysis of electrode materials through noble metals, metal alloys, transition metal oxides/hydroxides, or conductive polymers [1,5] assembled through both bottom-up and top-down, as well as mixed strategies, to avoid such issues [6]. Copper-based materials have been widely used to modify the electrode as a non-enzymatic sensor for glucose detection taking its low cost, suitable redox properties, and excellent stability into account [7]. A large variety of copper/copper oxides modified electrodes having various shapes and sizes of copper nanoparticles have been reported to date for the non-enzymatic detection of glucose [1,2,7,8,9,10,11,12,13,14,15,16,17,18,19]. Moreover, the role of the carbon substrate in enhancing the electrochemical performance of the copper-based nanostructured carbon electrode has been investigated [20,21,22]. Carbon nanomaterials have great potential in sensor applications due to their exceptional electrical, chemical, and mechanical properties. Carbon nanofibers (CNFs) are unique in the fact that their whole surface area can be activated. CNFs are cylindric nanostructures with graphene layers that are arranged as stacked cones, cups, or plates, with mechanical strength and electric properties being similar to carbon nanotubes while their size and graphite ordering can be well controlled [23]. They have been extensively used in electrochemical sensors and biosensors either as co-catalyst or/and catalyst support [9]. Indeed, they can be used both as immobilization matrixes for metal/metal oxides and as transducers due to their electrochemical signal [24,25,26].

Recently, the construction of micro-/nano-structures assembly-based electrode compositions containing copper/copper oxides has been studied for enhancing the electrocatalytic properties and implicit, the electroanalytical performance for the non-enzymatic detection of glucose through the one-pot solvothermal method [8] and the copper-based metal-organic framework [1]. 

Copper coordination complexes represent one of the largest classes containing copper with relevant photochemical and photophysical properties [27]. Inspired by the important role of copper in biological systems, biomimetic materials that were based on copper coordination complexes were obtained and reported as good catalysts for water oxidation [28] or oxygen reduction in fuel cells [29,30,31,32]. Cu(I) coordination complexes with chelating N^N ligands show appropriate photophysical and redox properties, being proposed as successful cheap and nontoxic alternatives in Light Emitting Devices (LEDs) instead of rare earth and noble metals [33] or Dye Sensitized Solar Cells (DSSCs) instead of Ru(II) [34]. The stability of Cu(I) species with N-donor ligands is highly dependent on the geometry environment of the metal centre, a tetracoordination that favours the low oxidation state, whereas higher coordination numbers facilitate oxidation to Cu(II) [35]. 2,2-biquinolines were found to form stable ionic Cu(I) coordination complexes due to the fused benzo-rings in the proximity of the chelating site, but the complexes still suffer from a significant degree of flattening of both ground and excited states, due to the presence of solvent or other coordinating moieties [36]. This sensitivity to the molecular environment might be used for the sensing and detection of various organic and biologic compounds. 

**Figure 1:**
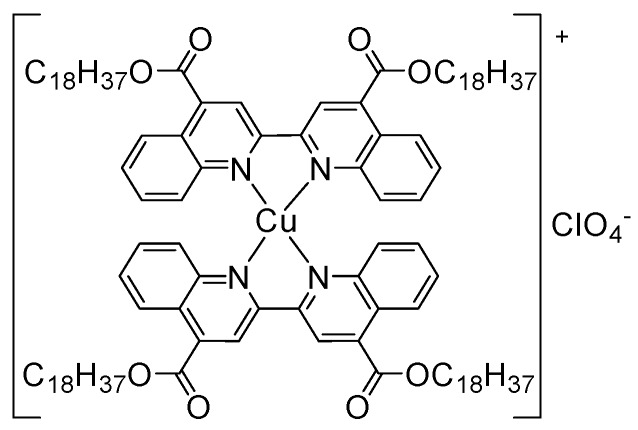
Chemical structure of the Cu(I)–C18 complex.

In this study, an ionic homoleptic Cu(I) coordination complex having the metal centre *bis*-chelated by 2,2′-biquinoline ligands that were functionalized in 4,4′-position with long alkyl chains (Cu(I)–C18) in Figure 1 was integrated within the composition of the carbon nanofibers combined with paraffin oil as paste electrode (Cu–C18/CNF). In-situ copper oxides are generated within the carbon nanofiber paste electrode (CuO*_x_*/CNF) for the non-enzymatic detection of glucose by electrochemical transformations in alkaline medium. 

The self-assembly of copper oxides into randomized microarrays should be achieved within carbon nanofiber paste electrode because of the structural characteristics of Cu(I) coordination complex. To the best of our knowledge, this is the first study on the use of an ionic Cu(I) coordination complex *bis*-chelated by N^N-donor ligands functionalized with long alkyl chains for obtaining randomized copper oxides microarray dispersed within carbon nanofiber paste electrode (CuO*_x_*/CNF) for enhanced glucose detection. The electroanalytical performance was assessed by it comparing with other commercial and reported copper oxides-based electrodes.

## 2. Materials and Methods

Cu(I)–C18 was prepared as previously reported [37]. The carbon nanofibers with a diameter ranged from 50–150 nm and purity higher than 98%, and paraffin oil that were used for electrode composition were of analytical standard, being provided by Sigma Aldrich (Steinheim, Germany). A Cary 630 FT-IR spectrophotometer was used to collect Fourier transform infrared spectroscopy (FTIR) spectra of Cu(I)–C18, Cu–C18/CNF, and CNF in paraffin oil paste at room temperature in the wavenumber range of 4000–400 cm^−1^ using transmission technique. A scanning electronic microscope (SEM, Inspect S PANalytical model) was used to characterize comparatively the morphological surfaces of Cu–C18/CNF, CuO*_x_*/CNF, and the simple carbon nanofiber paste electrode (CNF). A three-electrode cell consisting of a Cu–C18/CNF paste working electrode, a platinum counter electrode, and silver/silver chloride reference electrode (Ag/AgCl, KCl 3M) connected to an Autolab potentiostat/galvanostat PGSTAT 302 (Eco Chemie, The Netherlands) that was controlled with GPES 4.9 software was used for performing all of the electrochemical measurements. The disc geometry that was characterized by 0.01 cm diameter of the Cu–C18/CNF paste electrode was obtained by filling a Teflon mold. 0.1 M sodium hydroxide (NaOH) as the supporting electrolyte was used to assure the alkaline medium desired for in-situ obtaining of randomized copper oxides microarray dispersed within carbon naofiber paste electrode (CuO*_x_*/CNF) by electrode potential scanning. An electrochemical stabilization through 10 continuous repetitive cyclic voltammograms within the potential ranging between −0.5 and +1.5 V vs. Ag/AgCl was applied prior to the electrode use. NaOH used was analytical-grade reagent from Merck and 0.1 M glucose was prepared daily from analytical grade Merck reagents while using double distillate water. The serum glucose was obtained from Vioser S.A., Parenteral Solution Industry, Greece. Cyclic voltammetry (CV), differential pulsed voltammetry (DPV), square-wave voltammetry (SWV), chronoamperometry (CA), and multiple pulsed amperometry (MPA) were the electrochemical techniques that were applied for electrochemical characterization and analytical applications. 

The electroanalytical parameters, including as the limit of detection (LOD) and the limit of quantification (LOQ), were calculated as:

LOD = 3 × *SD*/*m* (1)

LOQ = 10 × *SD*/*m* (2)

where *SD* is the standard deviation of the six blanks and *m* is the slope of the calibration plot [24].

## 3. Results

The synthesis and characterization of the ionic homoleptic Cu(I) coordination complex (Cu(I)–C18) tested in the present work was previously reported [37]. Its chemical structure is presented in Figure 1. The complex was chosen for its structural characteristics: the bio metal centre is *bis*-chelated by two N^N-donor ligands that were functionalized with long alkyl chains that induce the complex liphophilicity and a waxy morphology that increases the loading on the carbon nanofiber in the electrode composition during preparation. The complex electric neutrality is given by ClO_4_ counterion.

### 3.1. Preparation of the Cu–Carbon Nanofiber Paste Electrode (Cu-C18/CNF) 

The Cu–C18/CNF paste electrode was obtained by mixing appropriated weights of carbon nanofibers, paraffin oil, and Cu(I)–C18 in order to obtain the ratio of 40%, wt. carbon nanofibers, 20%, wt. Cu(I)–C18 complex, and 40%, wt. paraffin oil. A blank carbon nanofiber paste electrode (CNF) was with the composition of 75 wt.% carbon nanofibers and 25 wt.% paraffin oil was analogously obtained. To assure the electrode stability and an adequate contribution of Cu(I) complex, the mass ratio of Cu(I)–C18: CNFs: paraffin oil of 1:2:2 was chosen., the ratio of 3:1 carbon nanofiber to paraffin oil as the carbon nanofiber paste was used for comparison.

### 3.2. Structural and Morphological Characterization 

**Figure 2:**
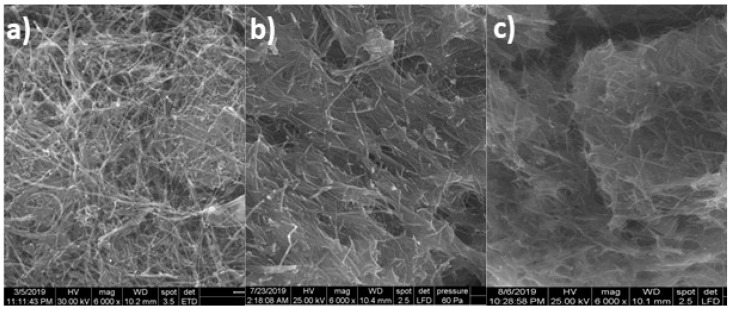
Scanning electron microscopy (SEM) images at 6000× magnification of (**a**) Cu–C18/CNF; (**b**) CNF; and, (**c**) CuO*_x_*/CNF.

The CuO*_x_*/CNF paste electrode was characterized morpho-structurally by scanning electron microscopy (SEM) after the electrochemical treatment of Cu–C18/CNF paste electrode by potential scanning through cyclic voltammetry (CV) in 0.1 M alkaline medium (Figure 2c). By comparing the results with the SEM images of Cu–C18/CNF (Figure 2a) and CNF paste electrodes (Figure 2b), it can be noticed the presence of Cu/Cu oxides in a good distribution and non-homogeneous dispersion onto CNFs mixed with paraffin oil.

**Figure 3:**
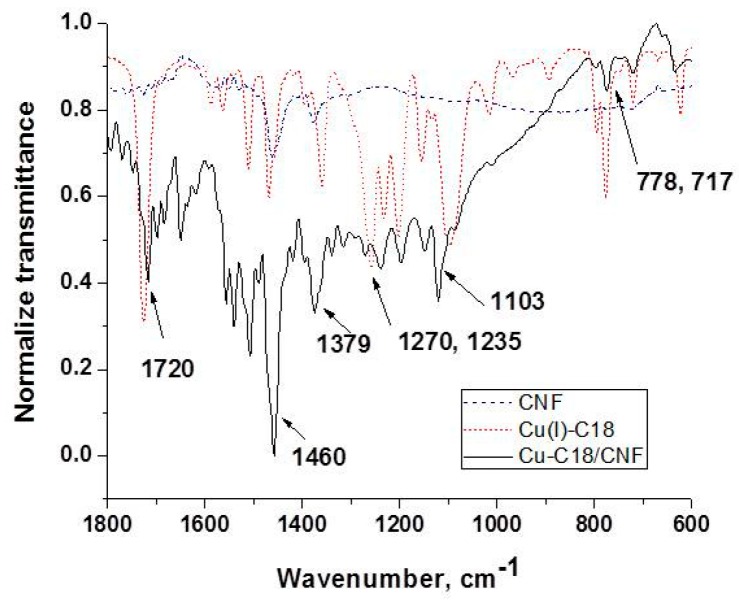
IR spectra of (1) CNF; (2) Cu(I)–C18; and (3) Cu–C18/CNF.

Figure 3 presents the IR spectra of the carbon nanofiber CNF, Cu(I) coordination complex Cu(I)–C18 and Cu–C18/CNF paste electrode in the representative region of 1800–600 cm^−1^. For CNF, the IR spectrum shows only the bands related to the paraffin oil, respectively, 1460, 1379, and 717 cm^−1^ assigned to the C–H bending vibration and in-plane deformation rocking vibration [38]. In the same region, the spectrum of Cu(I)–C18 contains additional intense vibrational bands assigned to C=O (1720 cm^−1^), in-plane, and out-of-plane aromatic C–H bending (1270 cm^−1^, 778 cm^−1^), C–O (1235 cm^−1^) stretching, and the characteristic band of ClO_4_^−^ counterion (1103 cm^−1^) [37]. Finally, the spectrum of Cu–C18/CNF shows the most intense vibrational bands of the Cu(I) complex proving the successful incorporation of the copper complex in the carbon nanofiber paste electrode. Besides this region, all of the spectra contain the C–H stretching vibration of CH_3_ and CH_2_ related to the paraffin oil and to the alkyl chains belonging to the Cu(I) coordination complex at 2845 and 2912 cm^−1^.

### 3.3. Electrochemical Characterization for Glucose Detection

The CNF paste, Cu–C18/CNF, and CuO*_x_*/CNF paste electrodes were analysed in terms of the electroactive area, which was determined while using the classical ferro/ferricyanide redox system. All the CNFs based paste electrodes exhibited about two-fold higher electroactive areas in comparison with the geometric ones (0.0270 cm^2^ vs. 0.0176 cm^2^).

**Figure 4:**
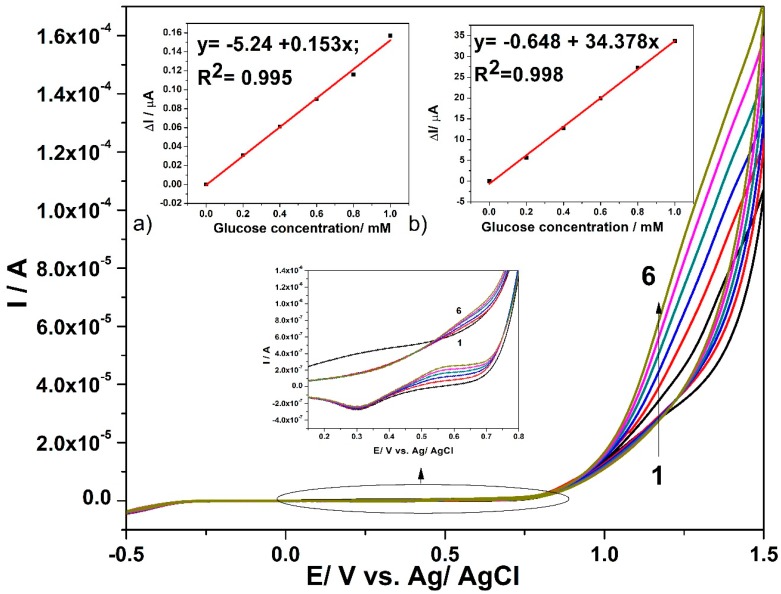
Cyclic voltammograms recorded with CuO*_x_*/CNF composite electrode in 0.1 M NaOH supporting electrolyte (curve 1) and in the presence of various glucose concentrations: 0.2 mM-curve 2; 0.4 mM-curve 3; 0.6 mM-curve 4; 0.8 mM-curve 5; 1 mM-curve 6; Inset: Calibration plots of the anodic current vs. glucose concentration, recorded at: (**a**) +0.6 V vs. Ag/AgCl (MSE* of 1.422 × 10^−5^ µA) and (**b**) +1.2 V vs. Ag/AgCl (MSE of 0.285 µA). (MSE* = mean square error value.)

CNF and in-situ formed CuO*_x_*/CNF paste electrodes were tested in 0.1 M NaOH and in the presence of various glucose concentrations while using cyclic voltammetry (CV). Figure 4 presents cyclic voltammograms recorded on CuO*_x_*/CNF paste electrode within the potential range from −0.5 V/ vs. Ag/AgCl to +1.5 vs. Ag/AgCl in the presence of glucose concentrations ranged from 0.2 mM to 1 mM. 

The presence of the anodic current peak at a potential value of +0.2 V/ vs. Ag/AgCl can be noticed, which can be attributed to the Cu(I)/Cu(II) redox system. In the presence of glucose, this peak decreases probably because of glucose adsorption on the Cu_2_O/CuO generated in the alkaline medium [16,39]. Starting with the electrode potential higher than +0.5 V vs. Ag/AgCl, the anodic current peak appears in the presence of glucose and linearly increases with its concentration within the tested concentration range, which is attributed to the oxidation process of glucose. According to the literature [16,39,40], the glucose oxidation process is catalysed by the presence of Cu(II)/Cu(III) species. Additionally, within the potential range of the oxygen evolution reaction that started under these working conditions at the potential value of about +1 V vs. Ag/AgCl, the anodic current linearly increases with the glucose concentration. The sensitivities for the glucose detection at CuO*_x_*/CNF paste electrode determined as slope of the linear plots of the anodic currents versus glucose concentrations of Figure 4a,b are 0.153 µA·mM^−1^ at the potential value of +0.6 V vs. Ag/AgCl and 34.378 µA·mM^−1^ at the potential value of +1.1 V vs. Ag/AgCl. A similar test was performed with CNF paste electrode and no response in the presence of glucose was detected (the results are not shown here). For glucose concentration higher than 1mM glucose, the sensitivities probably decrease because of the electrode fouling. Several mechanistic aspects related to the electrochemical behaviour of the CuO*_x_*/CNF paste electrode for designing the electrochemical detection method derive from the influence of the scan rate on the shapes of cyclic voltammograms in alkaline medium, in the absence and presence of 0.6 mM glucose, respectively. The specific micro-dispersed nature of Cu(I) that is responsible for copper oxides generation suggests a discussion about the typical aspects regarding the behaviour of CuO*_x_*/CNF electrode as randomized or ordered array structured copper-based microelectrodes system, which should assure the heterogeneous activity with the distinct micro zones of a significantly higher electrocatalytic activity [41]. The voltammetric response of Cu(I) dispersed and arranged through Cu(I)–C18 coordination complex within carbon nanofiber should be similar to that found for “edge effect” that significantly contribute to the Faradaic response. The size, shape, and inter-microelectrode separation are responsible for the microelectrode array behaviour. Closely spaced Cu(I) micro zones will behave similarly to a macroelectrode and the linear diffusion dominates the mass transport because of the diffusion layer overlap, which is in contrast with a microelectrode array characterized by spherical diffusion mass transport. A larger distance between microelectrodes also repeals the microelectrode array behaviour and individual microelectrode behaviour should be manifested [42,43,44]. It has been demonstrated that microelectrode arrays overcome the main drawbacks exhibited by single microelectrode related to low current outlet and high susceptibility to the electrochemical noise. At the same time, they maintain single microelectrode advantages regarding the minimization of the ohmic drop and charging current, allowing for lower detection limit and better sensitivity [45].

**Figure 5:**
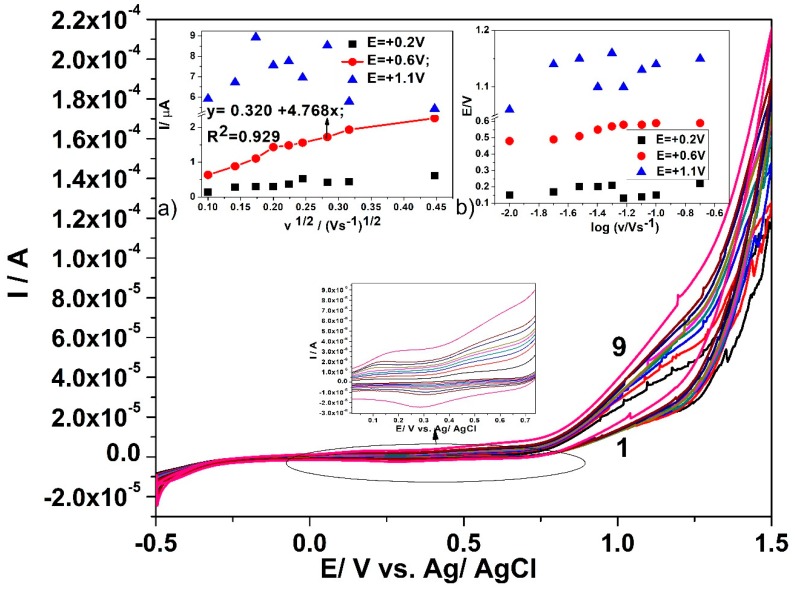
Cyclic voltammograms recorded in 0.6 mM glucose and 0.1 M NaOH supporting electrolyte with the CuO*_x_*/CNF paste electrode at various scan rates: (curve 1) 10, (curve 2) 20, (curve 3) 30, (curve 4) 40, (curve 5) 50, (curve 6) 60, (curve 7) 80, (curve 8) 100, and (curve 9) 200 mV·s^−1^. Insets: (**a**) dependence of anodic peak current vs. square root of the scan rate (MSE of.018 µA); (**b**) dependence of peak potential vs. logarithm of the scan rate.

Figure 5 illustrates the effects of the scan rate on the cyclic voltammograms recorded on CuO*_x_*/CNF paste electrode. The scan rate is systematically varied from 0.002 to 0.2 V·s^−1^ in the presence of 0.6 mM glucose in 0.1 M NaOH supporting electrolyte. The effect of the scan rate was also studied under similar working conditions in the absence of glucose to elucidate the kinetic aspects related to copper oxides that are involved in the electrochemical oxidation of glucose. The variation of the anodic current (with the background subtraction) vs. the square root of the scan rate in Figure 5a only shows at the potential value of +0.6 V vs. Ag/Ag/Cl a linear dependence corresponding to linear diffusion characteristics to the macroelectrode. At the potential value of +1.1 V vs. Ag/Ag/Cl, no linear dependence of the anodic current is found that might be explained rather due to a competition between oxygen evolution reaction and advanced glucose oxidation than a non-linear or spherical diffusion-controlled overall process that is characteristic to ordered microarray behaviour. Additionally, no linear dependence of the anodic peak current (with background subtraction) vs. the square root of the scan rate is found at the potential value of +0.2 V vs. Ag/AgCl, which is probably due to the involvement of surface-controlled step proving the glucose sorption on the copper oxides. These results show that the CuO*_x_*/CNF paste electrode acts rather as randomized than an ordered array of copper oxides microelectrode. The lack of the cathodic peaks corresponding to the anodic one’s characteristics to glucose oxidation (+0.6 V and respective, +1.1 V vs. Ag/AgCl) indicates the irreversibility of glucose oxidation, even if no linear dependence of oxidation potential values vs. the logarithm of the scan rate was noticed of Figure 5b).

Based on these observations that were correlated with the literature reported data [46], the main mechanism of glucose sensing at the potential value of +0.6 V vs. Ag/AgCl may be described by the following reactions:

Cu(I)_(complex)_ + OH^−^ → Cu(OH)_2_ + e^−^, (3)

Cu(OH)_2_ + OH^−^ → CuOOH + H_2_O + e^−^ (4)

CuOOH + e^−^ + glucose → CuO + OH^−^ + gluconolactone (5)

Gluconolactone + Cu(II) → gluconic acid (hydrolysis) + Cu(II)(6)

When the potential CuO value increases higher than +1.00 V/ vs. Ag/AgCl, the population of CuOOH sites on the electrode surface increases, and subsequent catalytic oxidation of gluconolactone occurs. Hydroxyl radicals that can be formed under this potential range also favours the latter. The mechanism of the glucose oxidation within the oxygen evolution reaction range has not yet been fully elucidated [14]. However, the presence of glycolate, oxalate, and formate has been reported for advanced electrooxidation of glucose through enediol intermediate [17,19]. Additionally, Luo and Baldwin [16] suggested that the main product of advanced electrooxidation of glucose on copper electrode is formate, beginning at C1 position. Additionally, low levels of gluconic, glucuronic, and glucaric acids derivates were reported as advanced glucose oxidation products through oxidation in C1 and C6 positions.

### 3.4. Detection Results

The above presented results of CV that were recorded at increased glucose concentrations indicate that the peaks corresponding to the glucose oxidation recorded at the potential values of +0.6 V vs. Ag/AgCl and +1.1 V vs. Ag/AgCl can be chosen for glucose detection. It is clear that a lower potential value is desired for sensing, but the better sensitivity reaching a higher potential value implies considering both detection potentials for optimization in relation with advanced voltammetric and amperometric techniques, e.g., differential-pulsed voltammetry (DPV), square-wave voltammetry (SWV), chronoameprometry (CA), and multiple-pulsed amperometry (MPA). 

It is well-known that the DPV and SWV techniques under optimized working conditions allow for a diminution of the capacitive component, while the Faradaic component of the anodic current corresponding to the glucose oxidation increases with the consequence of enhanced electroanalytical performance of certain electrode-based detection methods related to the lowest limit of detection, quantification, and the sensitivities [24]. It must be mentioned that the detection response stability is linked to the electrode composition, which limits the DPV and SWV working conditions.

**Figure 6:**
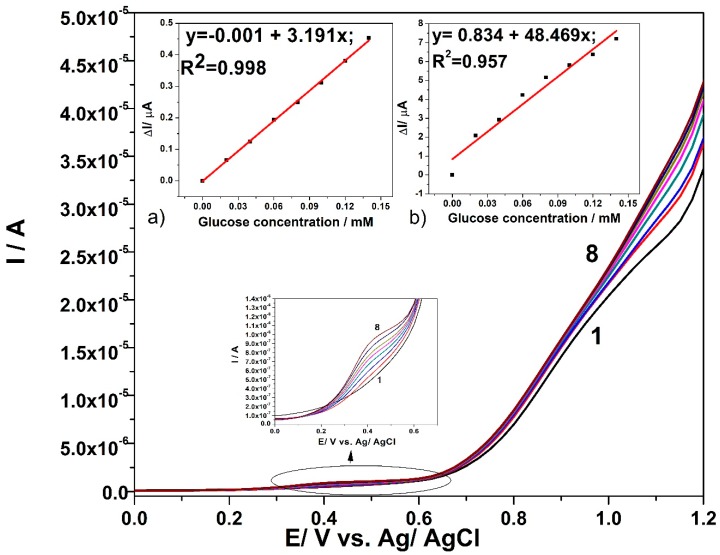
Differential pulsed voltammetry (DPV) responses in 0.1 M NaOH supporting electrolyte (curve 1) and in the presence of various glucose concentrations: 0.02 mM-curve 2; 0.04 mM-curve 3; 0.06 mM-curve 4; 0.08 mM-curve 5; 0.1 mM-curve 6; 0.12 mM-curve 7; 0.14 mM-curve 8, with CuO*_x_*/CNF paste electrode under operating conditions: MA of 0.1 V and SP of 0.025 V; Inset: Calibration plots of current vs. glucose concentration at the detection potential of: (**a**) +0.4 V vs. Ag/AgCl (MSE of 2.705 × 10^−5^ µA) and (**b**) +1.1 V vs. Ag/AgCl (MSE of 0.252 µA).

Figure 6 displays the optimized results of DPV technique applied to CuO*_x_*/CNF electrode in the presence of increased glucose concentration under optimized working conditions related to the modulation amplitude (MA) of 0.1 V and the step potential (SP) of 0.025 V. The detection responses that were obtained for the glucose concentration range lower by ten-fold than the one used for CV applying gives a first preliminary result related to the enhanced glucose concentration detection associated to the lowest limit of detection. The slopes of the calibration plots that are presented in insets of Figure 6, which give the sensitivities in glucose detection, show better sensitivities for both detection potential values, which are shifted to lower positive values (+0.4 V vs. Ag/AgCl as compared with +0.6 V vs. Ag/AgCl and +1.1 V vs. Ag/AgCl when compared with +1.2 V vs. Ag/AgCl) in comparison with CV. These results prove the superiority of the DPV technique in relation with the CV.

**Figure 7:**
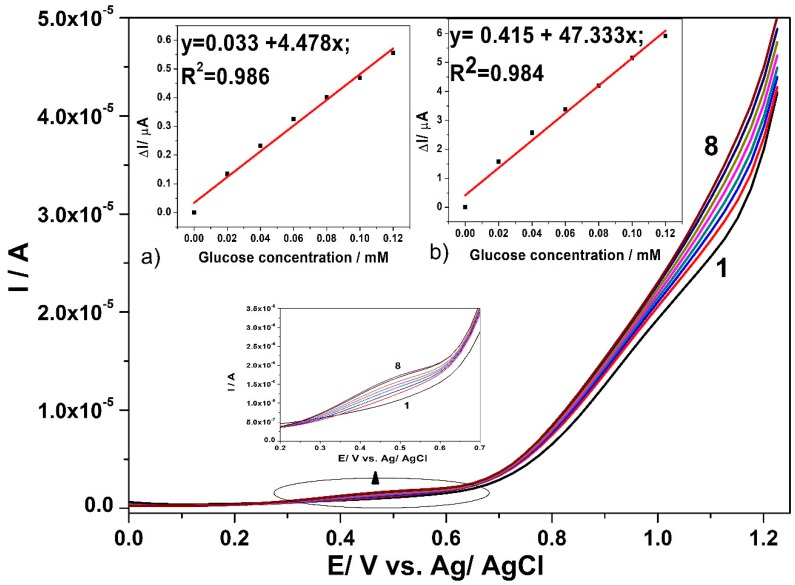
SWV responses recorded with CuO*_x_*/CNF paste electrode in 0.1 M NaOH supporting electrolyte (curve 1) and in the present of various glucose concentrations: 0.02 mM-curve 2; 0.04 mM-curve 3; 0.06 mM-curve 4; 0.08 mM-curve 5; 0.1 mM-curve 6; 0.12 mM-curve 7; 0.14 mM-curve 8, under operating conditions: step potential (SP) 0.025 V; modulation amplitude (MA) 0.1 V; frequency (*f*) 5Hz. Inset: Calibration plots of current vs. glucose concentration at the detection potential of: (**a**) +0.4 V vs. Ag/AgCl (MSE of 5.229 × 10^−4^ µA) and (**b**) +1.1 V vs. Ag/AgCl (MSE of 0.067 µA).

For comparison, the SWV technique was employed under optimized conditions that were found for DPV, i.e., MA of 0.1 V and the SP of 0.025 V at various values of frequency (*f*) ranged from 5 to 50 Hz. At a high frequency value, the electrode signal is not stable and the best results are found for the frequency value of 5 Hz, which Figure 7 presents. Good linearities of the current vs. glucose concentration are achieved at the potential value of +0.45 V vs. Ag/AgCl and +1.1 V vs. Ag/AgCl, presented in the Figure 7a,b. Slight improvement of the sensitivities determined as slopes of the calibration plots are achieved by SWV applying, in comparison with DPV.

**Figure 8:**
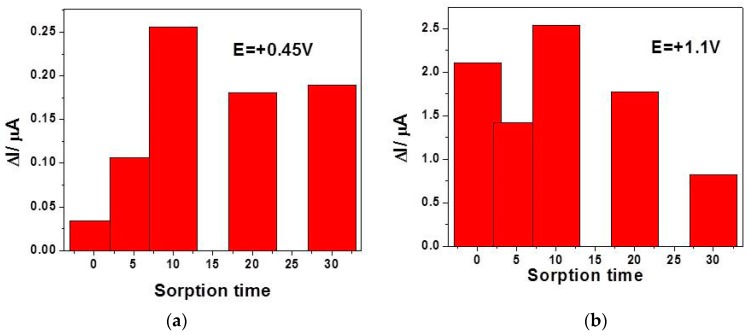
Useful signals achieved by square-wave voltammetry (SWV) recorded in the presence of 0.02 mM containing 0.1 M NaOH supporting electrolyte at CuO*_x_*/CNF electrode, as a function of the sorption time in the preconcentration step prior to detection recorded at: (**a**) E = +0.45 V vs. Ag/AgCl and (**b**) +1.1 V vs. Ag/AgCl.

While taking the step of glucose sorption onto copper oxides and the SVW technique abilities into consideration, a preconcentration step before SWV running and recording was tested for various sorption times considered as accumulation time to improve the electroanalytical parameters of the glucose detection. The preconcentration step consisted in the CuO*_x_*/CNF paste electrode immersion in low glucose concentration (0.02 mM) containing supporting electrolyte at the open circuit potential for various sorption times. Figure 8a,b gather the results of the current signal corresponding to the detection of 0.02 mM glucose at both detection potential values of +0.45 V vs. Ag/AgCl and +1.1 V vs. Ag/AgCl.

It can be noticed that the effect of the accumulation is highly manifested at the potential value of +0.45 V vs. Ag/AgCl for the first 10 min, while at the potential value of +1.1 V vs. Ag/AgCl a randomized effect is observed, probably due to the competition with oxygen evolution. The best signal is also found at 10 min accumulation time, which should be considered to be optimum. A longer accumulation time leads to a weakening of the useful signal for glucose detection, probably due to the fouling effect that begins to manifest. By applying preconcentration step (10 min) based SWV under the above-presented optimized conditions (0.1 V MA, 0.025 V SP, and frequency of 5 Hz), the electroanalytical parameters for the glucose detection are enhanced at both detection potential values (7.94 as compared with 4.48 µA·mM^−1^ for +0.45 V vs. Ag/AgCl and 95.40 compared with 47.33 µA·mM^−1^ for +1.1 V vs. Ag/AgCl).

**Figure 9:**
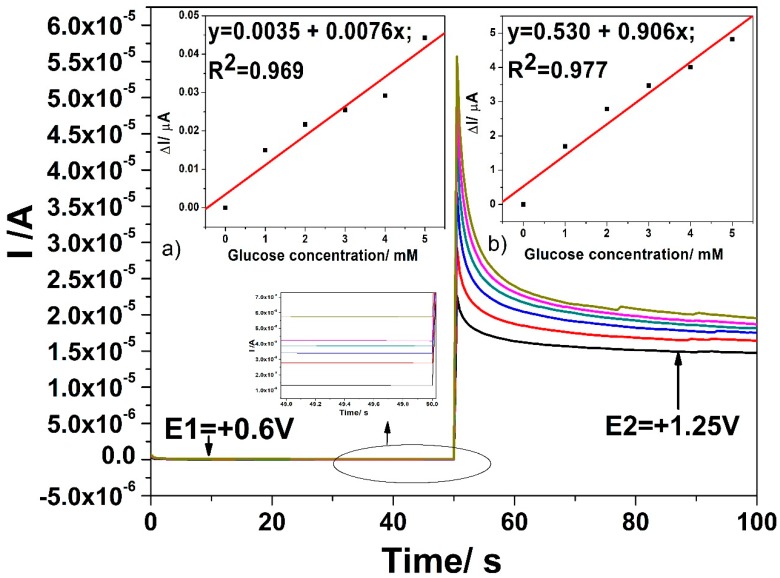
Chronoamperograms (CAs) recorded for two levels of detection potential, E1 = +0.6 V vs. Ag/AgCl and E2 = +1.25 V vs. Ag/AgCl, with the CuO*_x_*/CNF composite electrode in 0.1 M NaOH supporting electrolyte (black line) and in the presence of various glucose concentrations: 1 mM (red line); –2 mM (blue line); 3 mM (green line); 4 mM (pink line); 5 mM (lime green line). Inset: Calibration plots of current vs. glucose concentration at the detection potential of: (**a**) +0.6 V vs. Ag/AgCl (MSE of 1.659 × 10^−5^ µA) and (**b**) +1.25 V vs. Ag/AgCl (MSE of 0.098 µA).

The performance of the CuO*_x_*/CNF paste electrode was further tested by amperometric measurements while taking their simplicity and easy employing for real applications into consideration. It is obvious that the detection potential was selected based on the results of the cyclic voltammetry as reference. Figure 9 shows chronoamperograms recorded with the CuO*_x_*/CNF paste electrode under stationary conditions and two potential levels, one of +0.6 V vs. Ag/AgCl and the other of +1.25 V vs. Ag/AgCl.

Linear dependences are achieved for the useful current signals versus glucose concentrations for both detection potentials. The lowest sensitivity at the potential value of +0.6 V vs. Ag/AgCl is noticed, which the electrode fouling manifesting should explain. At the higher potential value of +1.25 V vs. Ag/AgCl better sensitivity was achieved but also lower in comparison with voltammetric results at the same potential value. It is clear that the electrode fouling occurred at both detection potential values leading to worse electroanalytical performance.

The multiple-pulsed amperometry (MPA) technique was applied under three potential-levels, while considering both the detection potential values and one level dedicated to renewing the electrode surface, to overcome the disadvantage of the electrode fouling during amperometric testing. This technique exhibits the possibility of in-situ cleaning and the refreshing of the electrode surface during detection measurements, combining the simplicity of amperometric technique with the advantage of the potential scanning at a certain potential corresponding to the desired electrode process that improved the electroanalytical performance. More variants of three potential-levels or two potential-levels were considered for MPA applying and the best results are reached for the variant that consisted of:

- +0.6 V vs. Ag/AgCl for a time duration of 50 ms, where glucose is oxidized to gluconolactone;
- +1.25 V vs. Ag/AgCl for a time duration of 100 ms, where glucose is further oxidized under oxygen evolution reaction generation; and,
- +0.75 V vs. Ag/AgCl for a time duration of 100 ms, to assure Cu(II)/Cu(III) system generation.

**Figure 10:**
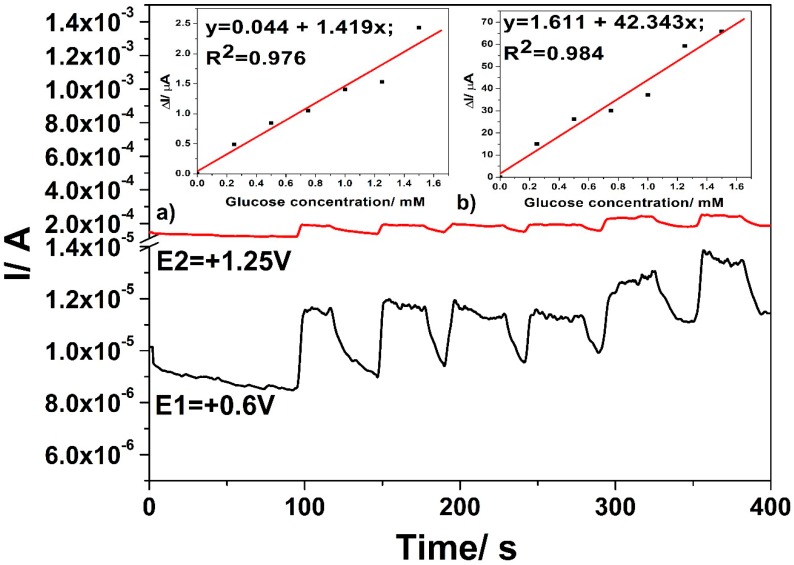
Multiple pulsed amperometries (MPAs) recorded for three levels of the potential pulses of +0.6 V vs. Ag/AgCl for 0.05 s (E1-black line), +1.25 V vs. Ag/AgCl for 0.1 s (E2-red line), and +0.75 V vs. Ag/AgCl for 0.1 s (E3, not shown here) with the CuO*_x_*/CNF composite electrode in 0.1 M NaOH supporting electrolyte and in the presence of various glucose concentrations: 0.25–1.25 mM; Insets: Calibration plots of the currents versus glucose concentrations at detection potential value: (**a**) +0.6 V vs. Ag/AgCl (MSE of 0.035 µA) and (**b**) +1.25 V vs. Ag/AgCl (MSE of 20.681 µA).

Figure 10 shows the MP amperograms responses after the successive addition of glucose in 0.1 M NaOH supporting electrolyte at each applied potential level and the insets of Figure 10 present the sensitivities determined as slopes of calibration plots of the useful current recorded at both detection potential values. Sensitivities that were comparable with the voltammetric techniques were achieved under the three potential-levels based MPA technique.

No linear dependence of the current signal versus glucose concentration was achieved at the potential value of +0.6 V vs. Ag/AgCl if the potential that was specific to electrode surface refreshing was set to a lower value, at which Cu(I) at least Cu(II) is generated (until +0.4 V vs. Ag/AgCl, the results are not shown here). This proved that the generation of Cu (II)/Cu(III) redox system, which is favoured at higher potential value, is responsible for the catalytic effect in glucose oxidation.

The results of the interference study for the CuO*_x_*/CNF paste electrode in the glucose detection while using optimized SWV in the presence of 0.1 mM uric acid and 0.1 mM ascorbic acid shows that the response recorded at +0.45 V vs. Ag/AgCl is affected while the response recorded at +1.1 V vs. Ag/AgCl is not affected. However, the electroanalytical performance of CuO*_x_*/CNF shows the best results at this higher potential value for both the voltammetric (SWV) and amperometric (MPA) technique (Table 1). The accuracy of the CuO*_x_*/CNF paste electrode was tested by the detection of glucose concentration in human serum, which was obtained from Vioser S.A. The current responses were recorded while using optimized SWV and series of SW voltammograms were achieved within various glucose concentrations from serum and the similar sensitivities were reached (the results are not shown here). The recovery test was performed for 0.5 mM and 1 mM glucose and the recovery percentage was 95% and, respectively, 105%, which shows the practical application potential of CuO*_x_*/CNF paste electrode for glucose detection in the biological sample. Three parallel electrodes were prepared and tested for similar 0.5 mM glucose concentration while using SWV under optimized conditions in order to assess the reproducibility of CuO*_x_*/CNF paste electrode. The relative standard deviation of 3.5% shows that the CuO*_x_*/CNF paste electrode exhibits a good reproducibility for glucose detection. The stability of the electrode was also examined measuring the current response to glucose over three months and about 5% loss in the current signal was found, which proves the good stability of the proposed electrode.

The performance of CuO*_x_*/CNF paste electrode is compared with other copper-based sensors reported in the literature for glucose detection (Table 2). It can be noticed that our proposed sensor exhibited the best sensitivity and the lowest limit of detection.

## 4. Conclusions

In summary, the Cu(I)–C18 complex–carbon nanofiber paste electrode (Cu–C18/CNF) was developed to prepare in-situ copper oxides within carbon nanofiber matrix in alkaline medium for glucose detection. By using a homoleptic ionic Cu(I) coordination complex that was based on 2,2′-biquinoline ligand functionalised with long alkyl chains, a randomized copper oxides microelectrodes array that was dispersed within carbon nanofiber paste (CuO*_x_*–CNF) was electrochemically obtained through potential scanning that ranged from −0.5 to +1.5 V vs. Ag/AgCl in 0.1 M NaOH solution using cyclic voltammetry (CV). Due to the structural characteristics of the Cu(I) complex, a good and non-homogeneous dispersion of copper/copper oxides within carbon nanofiber was found from the scanning electron microscopy (SEM) images. 

The CuO*_x_*/CNF paste electrode exhibited high electrocatalytic activity towards glucose oxidation manifested at two potential values of +0.6 V vs. Ag/AgCl and +1.2 V vs. Ag/AgCl, considered as a potential detection value for glucose in 0.1 M NaOH solution. The electroactive specific area of CuO*_x_*/CNF that was determined by classical ferri/ferrocyanide was two-fold higher than the geometrical one. All of the CV results involving the influence of the glucose concentration and the effect of the scan rate on the cyclic volatmmograms shapes in the presence of glucose proposed both detection potential values, +0.6 V vs. Ag/AgCl and +1.2 V vs. Ag/AgCl, which should be slightly modified in relation with the applied electrochemical technique. All of the tested electrochemical techniques, i.e., CV, DPV, SWV, CA, and MPA, allowed for glucose detection characterized by different electroanalytical parameters related to the sensitivity, the lowest limit of detection (LOD), and the lowest limit of quantification (LOQ). The optimized working conditions for detecting the glucose were found by using SWV as the voltammetric method and MPA as the amperometric detection method. Similar sensitivities of 2689.38 µA·mM^−1^·cm^−1^ and 2462.67 µA·mM^−1^·cm^−2^ were achieved for SWV-based voltammetric and MPA-based amperometric methods, which are much better that the reported copper-based electrodes. In addition, a preconcentration step involving 10 minutes accumulation at open circuit potential before SWV running allowed for further enhancing the sensitivity, 5419.77 µA·mM^−1^·cm^−1^ vs. 2689.38 µA·mM^−1^·cm^−1^ reached under the similar conditions, but without the preconcentration step. Based on the results of interference study, the electrode accuracy, stability and the life-time besides the excellent electrocatalytic properties for glucose oxidation, CuO*_x_*/CNF composition is expected as a promising electrode material for developing a non-enzymatic electrochemical glucose sensor.

## Figures and Tables

**Table 1:** The electroanalytical parameters for glucose direct detection with CuO*_x_*/CNF paste electrode in 0.1 M NaOH supporting electrolyte.

Tehnique	Potential Detection/ V vs. Ag/AgCl	Sensitivity/ µA·mM^−1^cm^−2^	Correlation Coefficient/R^2^	LOD^[a]^/µM	LQ^[a]^/µM	RSD^[b]^(%)
CV	+0.6 V	8.70	0.995	39	130	1.7
	+1.2 V	1953.30	0.998	1.88	6	1.5
DPV	+0.4 V	181.31	0.998	0.54	1.8	1.2
	+1.1 V	2753.92	0.957	0.07	0.23	0.5
SWV	+0.45 V	254.43	0.986	0.38	1.2	0.5
	+1.1 V	2689.38	0.984	0.07	0.24	1
Preconc./SWV	+0.45 V	451.25	0.979	0.43	1.45	1.6
	+1.1 V	5419.77	0.986	0.05	0.16	0.5
CA	+0.6 V	0.43	0.969	22	75	0.5
	+1.25 V	51.48	0.977	3.3	11	0.5
MPA	+0.6 V for 0.05 s	80.63	0.976	1.2	4.06	0.5
	+1.25 for 0.1 s	2462.67	0.984	0.082	0.27	0.5

^[a]^The lowest limit of detection (LOD) and the lowest limit of quantification (LQ) were determined according with the literature [47]. ^[b]^determined for three replicates.

**Table 2:** Comparison of performances of CuO*_x_*/CNF paste electrode with Cu-based electrode for non-enzymatic sensing of glucose.

Electrode Material	Linear Range (mM)	Supporting Electrolyte	Technique Used	Sensitivity (µA·mM^−1^·cm^−2^)	Detection Limit, µM	Reference
MOF-derived CuO arhitectures	0.0005–2.8	0.1 M NaOH	CA	934.2	0.1	[48]
CuO/CuBi_2_O_4_	0.1–8	0.1 M NaOH	CA	330	0.7	[49]
CuS microflowers	0.001–5.4	0.1 M NaOH	CA	1007	n.a.	[50]
CuO nanospheres	0.05–20	0.1 M NaOH	CA	404.53	1	[51]
CuO/graphene	5–14	Phosphate buffer (pH = 7.4)	CA	37.63	5	[9]
CuO/graphene	0.00021–12	0.1 M NaOH	CV	408.16	0.21	[9]
Cu-Cu_2_O nanoporous NPs	0.01–55	0.1 M NaOH	CA	123.8	0.05	[7]
Cu-Cu_2_O hollow microspheres	0.22–10.89	0.1 M NaOH	CA	33.63	0.05	[8]
Cu_2_O NPs	0.05–1.1	0.1 M NaOH	CA	53.69	47.2	[52]
3D Cu@Cu_2_O aerogels	0.001–17.2	0.1 M NaOH	CA	194.88	0.60	[53]
**CuO_x_/CNF**	0.02–0.14	0.1 M NaOH	Preconc.SWV/SWV	5419.7/2754^[a]^	0.048/0.07^[a]^	This work

^[a]^ without preconcentration step.

## References

[1] Kim K., Kim S., Lee H.N., Park Y.M., Bae Y.-S., Kim H.-J. (2019). Electrochemically derived CuO nanorod from copper-based metal-organic framework for non-enzymatic detection of glucose. Appl. Surf. Sci..

[2] Mohammadi S., Taheri A., Rezayati-zad Z. (2018). Ultrasensitive and selective non-enzymatic glucose detection based on pt electrode modified by carbon nanotubes@ graphene oxide/nickel hydroxide-Nafion hybrid composite in alkaline media. Prog. Chem. Biochem. Res..

[3] Campana A.L., Florez S.L., Noguera M.J., Fuentes O.P., Puentes P.R., Cruz J.C., Osma J.F. (2019). Enzyme-Based Electrochemical Biosensors for Microfluidic Platforms to Detect Pharmaceutical Residues in Wastewater. Biosensors.

[4] Chen C., Xie Q., Yang D., Xiao H., Fu Y., Tan Y., Yao S. (2013). Recent advances in electrochemical glucose biosensors: A review. RSC Adv..

[5] Park S., Boo H., Chung T.D. (2006). Electrochemical non-enzymatic glucose sensors. Anal. Chim. Acta.

[6] Calandra P., Caschera D., Liveri V.T., Lombardo D. (2015). How self-assembly of amphiphilic molecules can generate complexityin the nanoscale. Colloids Surf. A.

[7] Zhao Y.X., Li Y.P., He Z.Y., Yan Z.F. (2013). Facile preparation of Cu-Cu_2_O nanoporous nanoparticles as a potential catalyst for non-enzymatic glucose sensing. RSC Adv..

[8] Wang A.J., Feng J.J., Li Z.H., Liao Q.C., Wang Z.Z., Chen J.R. (2012). Solvothermal synthesis of Cu/Cu_2_O hollow microspheres for non-enzymatic amperometric glucose sensing. CrystEngComm.

[9] Foroughi F., Rahsepar M., Hadianfard M.J., Kim H. (2017). Microwave-assisted synthesis of graphene modified CuO nanoparticles for voltammetric enzyme-free sensing of glucose at biological pH values. Microchim. Acta.

[10] Raziq A., Tariq M., Hussain R., Mehmood M.H., Ullah I., Khan J., Muhammad M. (2018). Highly sensitive, non-enzymatic and precious metal-free electrochemical glucose sensor based on a Ni-Cu/TiO_2_ modifiedglassy carbon electrode. J. Serb. Chem. Soc..

[11] Yu G., Zhang W., Zhao Q., Wu W., Wei X., Lu Q. (2016). Enhancing the sensitivity of hexachlorobenzene electrochemicalsensor based on nitrogen–doped graphene. Sens. Actuat. B-Chem..

[12] Bott A.W. (1998). Electrochemical Methods for the Determination of Glucose. Curr. Sep..

[13] Chung R.-J., Wang A.-N., Liao Q.-L., Chuang K.-Y. (2017). Non-Enzymatic Glucose Sensor Composed of Carbon-Coated Nano-Zinc Oxide. Nanomaterials.

[14] Sakamoto M., Takamura K. (1982). 522—Catalytic oxidation of biological components on platinum electrodes modified by adsorbed metals. Anodic oxidation of glucose. Bioelectrochem. Bioenerg..

[15] Wang J., Cao X., Wang X., Yang S., Wang R. (2014). Electrochemical Oxidation and Determination of Glucose in Alkaline Media based on Au (111)-like Nanoparticle Array on Indium Tin Oxide Electrode. Electrochim. Acta.

[16] Luo M.Z., Baldwin R.P. (1995). Characterization of carbohydrate oxidation at copper electrodes. J. Electroanal. Chem..

[17] Larew L.A., Johnson D.C. (1989). Concentration dependence of the mechanism of glucose oxidation at gold electrodes in alkaline media. J. Electroanal. Chem..

[18] Karra S., Wooten M., Griffith W., Gorski W. (2016). Morphology of Gold Nanoparticles and Electrocatalysis of Glucose Oxidation. Electrochim. Acta.

[19] Tominaga M., Shimazoe T., Nagashima M., Taniguchi I. (2005). Electrocatalytic oxidation of glucose at gold nanoparticle-modified carbon electrodes in alkaline and neutral solutions. Electrochem. Commun..

[20] Pop A., Manea F., Orha C., Motoc S., Ilinoiu E., Vaszilcsin N., Schoonman J. (2012). Copper-decorated carbon nanotubes-based composite electrodes for nonenzymatic detection of glucose. Nanoscale Res. Lett..

[21] Liu M., Liu R., Chen W. (2013). Graphene wrapped Cu2O nanocubes: Non-enzymatic electrochemical sensors for the detection of glucose and hydrogen peroxide with enhanced stability. Biosens. Bioelectron..

[22] Manea F., Motoc S., Pop A., Remes A., Schoonman J. (2012). Silver functionalized carbon nanofiber composite electrodes for ibuprofen detection. Nanoscale Res. Lett..

[23] Vamvakaki V., Tsagaraki K., Chaniotakis N. (2006). Carbon Nanofiber-Based Glucose Biosensor. Anal. Chem..

[24] Motoc S., Manea F., Orha C., Pop A. (2019). Enhanced Electrochemical Response of Diclofenac at a Fullerene-Carbon Nanofiber Paste Electrode. Sensors.

[25] Mugweru A., Mahmud R., Ghosh K., Wanekaya A.K. (2017). Carbon nanofiber modified with osmium based redox polymer for glucose sensing. J. Electrochem. Sci. Eng..

[26] Zhang X., Liu D., Li L., You T. (2015). Direct Electrochemistry of Glucose Oxidase on Novel Free-Standing Nitrogen-Doped Carbon Nanospheres@Carbon Nanofibers Composite Film. Sci. Rep..

[27] Armaroli N., Accorsi G., Cardinali F., Listorti A. (2007). Photochemistry and Photophysics of Coordination Compounds: Copper. Top. Curr. Chem..

[28] Fengzhao T.S., Xia N. (2018). Metal Complexes as Molecular Electrocatalysts for Water Oxidation: A Mini-Review. Int. J. Electrochem. Sci..

[29] Zhao Y.-M., Yu G.-Q., Wang F.F., Wei P.-J., Liu J.-G. (2019). Bioinspired Transition-Metal Complexes as Electrocatalysts for the Oxygen Reduction Reaction. Chem. Eur. J..

[30] Gewirth A.A., Varnell J.A., DiAscro A.M. (2018). Nonprecious Metal Catalysts for Oxygen Reduction in Heterogeneous Aqueous Systems. Chem. Rev..

[31] Thorseth A., Tornow C.E., Tse E.C.M., Gewirth A.A. (2013). Cu complexes that catalyze the oxygen reduction reaction. Coord. Chem. Rev..

[32] Fukuzumi S., Lee Y.-M., Nam W. (2018). Mechanisms of Two-Electron versus Four-Electron Reduction of Dioxygen Catalyzed by Earth-Abundant Metal Complexes. ChemCatChem.

[33] Cariati E., Lucenti E., Botta C., Giovanella U., Marinotto D., Righetto S. (2016). Cu(I) hybrid inorganic–organic materials with intriguing stimuli responsive and optoelectronic properties. Coord. Chem. Rev..

[34] Ilmi R., Al-Busaidi I.J., Haque A., Khan M.S. (2018). REVIEW: Recent progress in coordination chemistry, photo-physical properties and applications of pyridine-based Cu(I) complexes. J. Coord. Chem..

[35] Crispini A., Cretu C., Aparaschivei D., Andelescu A.A., Sasca V., Badea V., Aiello I., Szerb E.I., Costisor O. (2018). Influence of the counterion on the geometry of Cu(I) and Cu(II) complexes with 1,10-phenanthroline. Inorg. Chim. Acta.

[36] Fransted K.A., Jackson N.E., Zong R., Mara M.W., Huang J., Harpham M.R., Shelby M.L., Thummel R.P., Chen L.X. (2014). Ultrafast Structural Dynamics of Cu(I)-Bicinchoninic Acid and Their Implications for Solar Energy Applications. J. Phys. Chem. A.

[37] Cretu C., Andelescu A.A., Candreva A., Crispini A., Szerb E.I., La Deda M. (2018). Bisubstituted-biquinoline Cu(I) complexes: Synthesis, mesomorphism and photophysical studies in solution and condensed states. J. Mater. Chem. C.

[38] Nie C., Tong X., Wu S., Gong S., Peng D. (2015). Paraffin confined in carbon nanotubes as nanoencapsulated phase change materials: Experimental and molecular dynamics studies. RSC Adv..

[39] Marioli J.M., Kuwana T. (1992). Electrochemical characterization of carbohydrate oxidation at copper electrodes. Electrochim. Acta.

[40] Manea F., Radovan C., Schoonman J. (2006). Amperometric determination of thiourea in alkaline media on a copper oxide–copper electrode. J. Appl. Electrochem..

[41] Ramirez-Garcia S., Alegret S., Cespedes F., Forster R.J. (2002). Carbon composite electrodes: Surface and electrochemical properties. Analyst.

[42] Stulik K., Amatore C., Holub K., Marecek V., Kutner W. (2000). Microelectrodes. Definitions, characterization and applications. Pure Appl. Chem..

[43] Simm A.O., Banks C.E., Ward-Jones S., Davies T.J., Lawrence N.S., Jones T.G.J., Jiang L. (2005). Boron-doped diamond microdisc arrays: Electrochemical characterisation and their use as a substrate for the production of microelectrode arrays of diverse metals (Ag, Au, Cu) via electrodeposition. Analyst.

[44] Davies T.J., Compton R.G. (2005). The cyclic and linear sweep voltammetry of regular and random arrays of microdisc electrodes: Theory. J. Electroanal. Chem..

[45] Cieciwa A., Wuthrich R., Comninellis C. (2006). Electrochemical characterization of mechanically implanted boron-doped diamond electrodes. Electrochem. Commun..

[46] Na W., Lee J., Jun J., Kim W., Kim Y.K., Jang J. (2019). Highly sensitive copper nanowire conductive electrode for nonenzymatic glucose detection. J. Ind. Eng. Chem..

[47] Konopka S.J., McDuffie B. (1970). Diffusion coefficients of ferri- and ferrocyanide ions in aqueous media, using twin-electrode thin-layer electrochemistry. Anal. Chem..

[48] Li L., Liu Y., Jiang J. (2019). Synthesis of the crystalline porous copper oxide architectures derived from metal-organic framework for electrocatalytic oxidation and sensitive detection of glucose. J. Ind. Eng. Chem..

[49] Wu C.-H., Onno E., Lin C.-Y. (2017). CuO nanoparticles decorated nano-dendrite-structured CuBi2O4 for highly sensitive and selective electrochemical detection of glucose. Electrochim. Acta.

[50] Radhakrishnan S., Ki H.-Y., Kim B.-S. (2016). A novel CuS microflower superstructure based sensitive and selective nonenzymatic glucose detection. Sens. Actuators B.

[51] Reitz E., Jia W., Gentile M., Wang Y., Lei Y. (2008). CuO Nanospheres Based Nonenzymatic Glucose Sensor. Electroanalysis.

[52] Li S., Zheng Y., Qin G.W., Ren Y., Pei W., Zuo L. (2011). Enzyme-free amperometric sensing of hydrogen peroxide and glucose at a hierarchical Cu2O modified electrode. Talanta.

[53] Gao Y., Yang F., Yu Q., Fan R., Yang M., Rao S., Lan Q., Yang Z., Yang Z. (2019). Three-dimensional porous Cu@Cu2O aerogels for direct voltammetric sensing of glucose. Microchim. Acta.

